# Enhanced Photocatalytic Performance of Nitrogen-Doped TiO_2_ Nanotube Arrays Using a Simple Annealing Process

**DOI:** 10.3390/mi9120618

**Published:** 2018-11-24

**Authors:** Phuoc Huu Le, Le Trung Hieu, Tu-Ngoc Lam, Nguyen Thi Nhat Hang, Nguyen Van Truong, Le Thi Cam Tuyen, Pham Thanh Phong, Jihperng Leu

**Affiliations:** 1 Ceramics and Biomaterials Research Group, Advanced Institute of Materials Science, Ton Duc Thang University, Ho Chi Minh City 700000, Vietnam; 2 Faculty of Applied Sciences, Ton Duc Thang University, Ho Chi Minh City 700000, Vietnam; 3 Department of Materials Science and Engineering, National Chiao Tung University, Hsinchu 30049, Taiwan; letrunghpc@gmail.com (L.T.H.); lamtungoc1310@gmail.com (T.-N.L.); 4 Faculty of Natural Sciences, Thu Dau Mot University, 6 Tran Van On Street, Thu Dau Mot City 820000, Vietnam; hangntn@tdmu.edu.vn; 5 Faculty of Fundamental Sciences, Thai Nguyen University of Technology, Thai Nguyen 24000, Vietnam; truonglyk3@gmail.com; 6 Laboratory of Magnetism and Magnetic Materials, Advanced Institute of Materials Science, Ton Duc Thang University, Ho Chi Minh City 700000, Vietnam; phamthanhphong@tdtu.edu.vn

**Keywords:** N-doped TNAs, two-step anodization, photocatalytic activity, thermal annealing, modified TiO_2_, band gap

## Abstract

Nitrogen-doped TiO_2_ nanotube arrays (N-TNAs) were successfully fabricated by a simple thermal annealing process in ambient N_2_ gas at 450 °C for 3 h. TNAs with modified morphologies were prepared by a two-step anodization using an aqueous NH_4_F/ethylene glycol solution. The N-doping concentration (0–9.47 at %) can be varied by controlling N_2_ gas flow rates between 0 and 500 cc/min during the annealing process. Photocatalytic performance of as-prepared TNAs and N-TNAs was studied by monitoring the methylene blue degradation under visible light (λ ≥ 400 nm) illumination at 120 mW·cm^−2^. N-TNAs exhibited appreciably enhanced photocatalytic activity as compared to TNAs. The reaction rate constant for N-TNAs (9.47 at % N) reached 0.26 h^−1^, which was a 125% improvement over that of TNAs (0.115 h^−1^). The significant enhanced photocatalytic activity of N-TNAs over TNAs is attributed to the synergistic effects of (1) a reduced band gap associated with the introduction of N-doping states to serve as carrier reservoir, and (2) a reduced electron‒hole recombination rate.

## 1. Introduction

Titanium dioxide (TiO_2_) is one of the most widely studied materials for applications in solar cells [1,2,3], pollutant degradation [4,5,6], photolysis of water [7], gas sensor [8], and bio-applications [9,10], due to its excellent photocatalytic activity, non-toxicity, high stability, low cost, and biocompatibility [11,12,13]. However, TiO_2_ is generally active under ultraviolet irradiation due to the wide band gap of approximately 3.2 eV [14,15], and has limited applications under visible light irradiation [16]. Thus, considerable effort has been made to improve the light absorption of TiO_2_ towards visible light via several schemes [17,18]: decorating TiO_2_ with noble metals [19], and doping TiO_2_ with noble metals [17] or non-metals (N, F, S) [20,21]. The studies of metal-doped TiO_2_ and noble metal-decorated TiO_2_ have demonstrated significant improvements in the photocatalytic performance of TiO_2_ under visible light irradiation [14]. However, these approaches showed several drawbacks such as thermal instability of doped TiO_2_, electron trapping by the metal centers, requirement of expensive ion-implantation facilities, and the use of precious metals. Alternatively, the substitution of anion species (i.e., C, N, F, P, S) in the anatase TiO_2_ crystal can induce a band gap narrowing effect [11,14,22]. Compared to other nonmetals, nitrogen doping is more appropriate for extending the photoactive region to visible light because of its comparable atomic size with oxygen, small ionization energy, metastable center formation, high stability, and low cost [23,24]. Theoretical calculations have also found that the p state of N is the most promising for substitutional doping into TiO_2_ and contributes to the band gap narrowing by mixing with O2p [14,21].

TiO_2_ nanomaterials are of great interest because of their large surface area and high light absorption capability [25,26,27,28,29]. Compared to other nanostructures, TiO_2_ nanotube arrays (TNAs) are of interest because they can provide a large surface-to-volume ratio and unidirectional electrical channel [30,31]. Several approaches to incorporate nitrogen into TNAs include one-step direct electrochemical anodization of a TiN alloy [32,33], anodization in the nitrogen-containing electrolyte [34], immersing TNAs in a N-containing solution, and performing post-annealing treatment [4,30,35]. It was found that N-doping was successfully achieved by thermal annealing of the as-prepared anodic TNAs in pure NH_3_ gas [36]. In addition, N-doped TiO_2_ thin films were prepared by sputtering in N_2_ gas containing ambient [37,38,39]. Jeon et al. [29] successfully developed three-dimensional nanostructured N-doped TiO_2_ monolith with enhanced visible light absorption, where the N-doping was obtained by thermal annealing of as-prepared 3D nanostructured TiO_2_ with a thin TiN layer coating on the surface. Recently, highly ordered TNAs with diverse top-layer morphology and enhanced photoelectrochemical and photocatalytic activity were obtained using the two-step anodization [31,40,41,42,43,44]. Based on these findings in the literature, we postulate that the synergetic effects of N-doping, modified morphology, and high-crystallinity TNAs can be achieved by using simple thermal annealing under controlled N_2_ gas flows. 

In this work, the surface N-doped TiO_2_ nanotube arrays (TNAs) with various doping concentrations from 0 to 9.47 at % were successfully achieved by a facile annealing process at 450 °C for 3 h and under different N_2_ gas flows between vacuum and 500 cc/min. The TNAs in this study were highly-ordered TNAs with a grid-like capping top layer, which were successfully prepared by a two-step anodization method using an NH_4_F and glycerol−water electrolyte. Structural–morphological, optical properties, and photocatalytic performance of the pristine and N-doped TNAs in the degradation of methylene blue under visible light irradiation (λ ≥ 400 nm) were studied and discussed in detail.

## 2. Materials and Methods

Titanium foil (99.9% purity, 1 cm × 2.5 cm size, 0.4 mm thickness) was used as the substrate for forming TNAs with modified morphologies by a two-step anodic oxidation. Prior to anodization, titanium (Ti) foil was ultrasonically cleaned in acetone, methanol and deionized water (each solvent 10 min), and then dried by a purging N_2_ gas. The anodization was carried out using a two-electrode system with the Ti foil as an anode and a stainless steel foil (SS304) as a cathode. All the electrolytes consisted of 0.3 wt % NH_4_F (SHOWA, Tokyo, Japan) in ethylene glycol solution with 2 vol % water. In the first-step anodization, Ti foil was anodized at 50 V for 1 h to form a nanotube layer. Then, the as-grown layer was ultrasonically removed for approximately 15 min until a bright surface appeared. The same Ti foil underwent the second anodization at 40 V for 30 min to form TNAs with a grid-like top-layer structure on the Ti substrates. The prepared sample was then immersed in ethanol solution for 5 h to remove the ethylene glycol contamination. Nitrogen-doping for the prepared TNAs were carried out by thermal annealing process at 450 °C for 3 h under various nitrogen flows: vacuum (~7 Pa), 200, 350, and 500 cc/min. Prior to introducing pure N_2_ gas (purity 99.99%), the annealing chamber was evacuated to a pressure of approximately 7 Pa for preventing contamination.

The crystal structures of the pristine and N-doped TNAs were characterized by X-ray diffraction (XRD, Bruker D2, Bruker, Billerica, MA, USA) using Cu Kα radiation (λ = 1.5406 Å) and high-resolution transmission electron microscopy (TEM, JEOL JEM-ARM200F, JEOL Ltd., Tokyo, Japan), operated at 200 kV. The TEM specimen was prepared by crashing the TNAs on TEM grids. X-ray photoelectron spectroscopy (XPS) experiments were conducted at beamline 09A2, NSRRC, Taiwan to determine the chemical bonding and surface N-doping concentration. XPS curve were fitted using the freeware XPSPEAK4.1 with the Shirley background subtraction and assuming a Gaussian‒Lorentzian peak shape. Morphologies of the pristine and N-doped TNAs were characterized by scanning electron microscopy (SEM, JEOL JSM-6500, JEOL Ltd.). Fundamental information on the energy levels lying within the band gap and charge carrier trapping and transfer was gathered by photoluminescence (PL) spectra, measured at room temperature (~22 °C). The excitation light source of the PL spectroscopy was an He‒Cd laser with a wavelength of 325 nm. The PL signal was dispersed by a Horiba Jobin Yvon IHR-320 single-grating (1800 grooves/nm grating) spectrometer (Horiba, Kyoto, Japan). The performance of pristine and N-doped TNAs was characterized by ultraviolet photoelectron spectroscopy (UPS) using monochromatized He-I radiation at 21.2 eV. The electrochemical impedance spectroscopy (EIS) measurements were performed using an Autolab system with a scan rate of 50 mV·s^−1^ and a conventional three-electrode test cell. The counter electrode was a platinum sheet, the reference electrode was Ag/AgCl (aqueous 3 M KCl), and TNAs and N-TNAs samples were used as a working electrode. The electrolyte was 0.1 M Na_2_SO_4_ aqueous solution under room light irradiation. 

The photocatalytic activity in the degradation of methylene blue (MB) of the samples was measured under visible light illumination at 120 mW·cm^−2^. The samples were illuminated using a Xenon lamp with a band-pass filter for λ ≥ 400 nm. Prior to illumination, the suspension was magnetically stirred for 20 min in the dark to ensure absorption‒desorption equilibrium between the photocatalyst and MB solution. The reaction temperature was kept at 32–33 °C for all samples. After a certain photocatalytic reaction time, the solution was taken for performing UV-Vis absorption spectrum to measure the MB concentration change using the characteristic peak at 654 nm.

## 3. Results and Discussion

**Figure 1:**
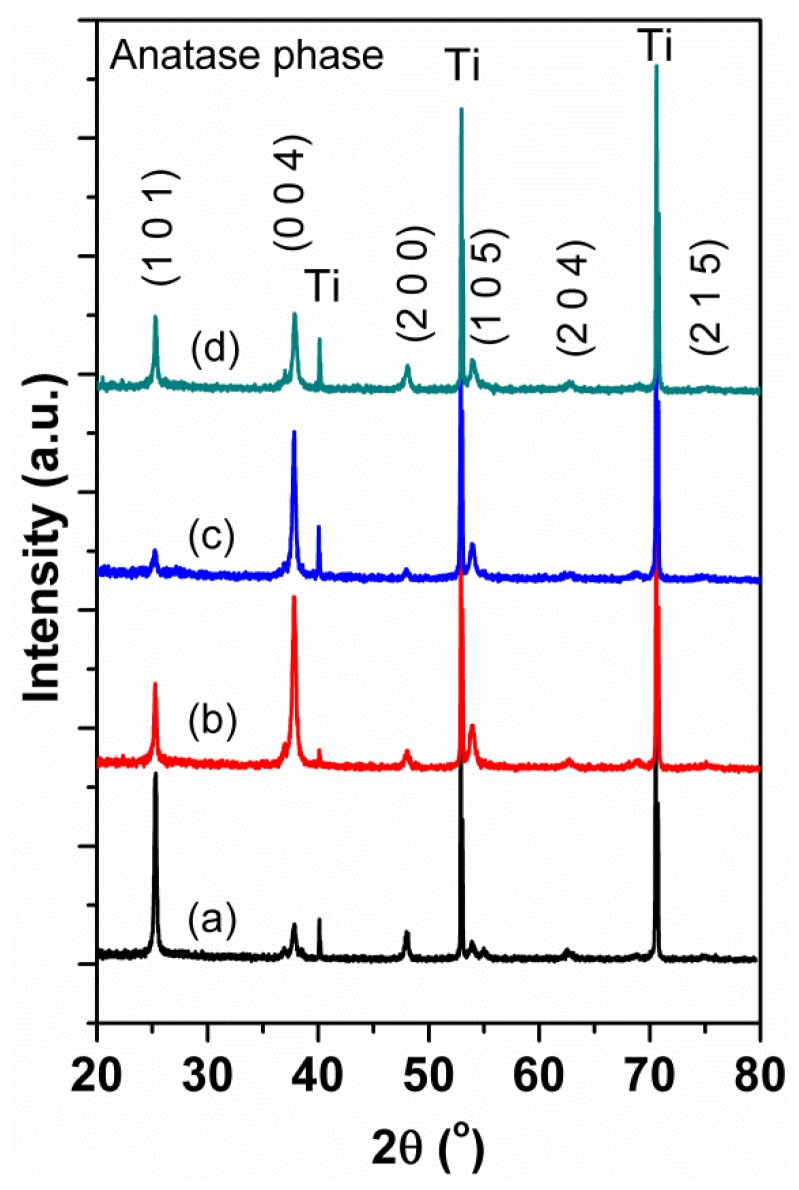
The XRD patterns of the pristine TNAs and N-doped TNAs with various N concentrations prepared by thermal annealing under different N_2_ gas flows: (**a**) 0 at % N in vacuum, (**b**) 5.76 at % N at 200 cc/min, (**c**) 9.47 at % N at 350 cc/min, and 6.60 at % N at 500 cc/min.

Figure 1 shows the XRD patterns of the pristine TNAs and N-doped TNAs with various nitrogen contents (see Table 1). All the samples presented the peaks at approximately 25.1°, 37.8°, 47.7°, 53.8°, 62.6°, and 75.1° corresponding to (101), (004), (200), (105), (204), and (215) faces of an anatase TiO_2_ phase (JCPDS No. 21–1272). Also, there were no rutile peaks, indicating a pure anatase phase for the TNAs in this study. The XRD patterns of the TNAs and N-TNAs are similar, agreeing with those reported in the literature [4,5,13,22,24]. Notably, the preferred orientation changed from (101) for TNAs to (004) for N-TNAs. The reason is not clear at present, but the change in preferred orientation to (004) for N-TNAs should not be due to N atom incorporation, which usually coincides with the shift of the XRD peak [23,45]; meanwhile, the (004)-dominant orientation was also found in TNAs (without N-doping) prepared by one-step or two-step anodization [43]. Recently, the intrinsic electric fields were found to be responsible for the oriented self-assembly of multilevel branched rutile-type TiO_2_ structures [46]. Therefore, for the present case, the unintentional slight difference in electric fields during anodic oxidation processes may play a role in determining the preferred orientation, i.e., (101) or (004).

**Figure 2:**
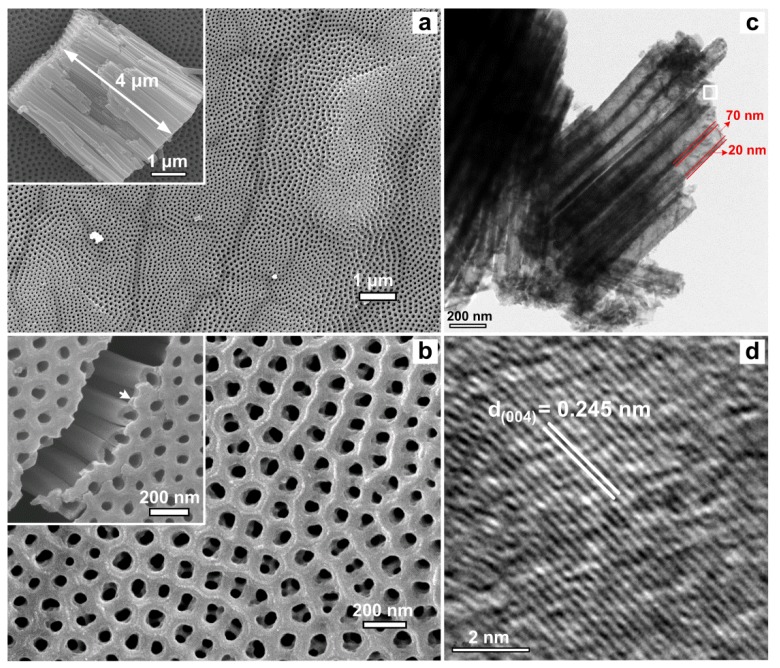
(**a**,**b**) SEM images of a typical N-doped TiO_2_ nanotube arrays (TNAs). The inset in (**a**) is a cross-sectional view of a TNA block. The inset in (**b**) shows grid-like top-layer covering on the TNAs. TEM images of a N-TNAs (9.47 at %): (**c**) a low magnification image at a TNA segment, (**d**) a high magnification image at the square area in (**c**).

Figure 2a,b show a typical morphology of N-doped TNAs. Clearly, the TNAs exhibit a highly ordered, uniformed, and clean surface. The thickness of the TNAs was approximately 4 µm, as shown in the inset of Figure 2a. The inset of Figure 2b shows a grid-like top layer, which plays a role as a capping layer to protect the TNAs’ structure from bundling and crumpling. Figure 2c,d shows TEM images of typical N-doped TNAs (i.e., 9.47 at %). The TNAs have an average tube diameter of ~70 nm and a wall thickness of ~20 nm. In addition, the lattice fringes with a *d*-spacing of 0.245 nm can be assigned to the (004) lattice plane of anatase TiO_2_ (Figure 2d). The (004)-lattice fringes were primarily observed from HRTEM images, further confirming the (004)-preferred orientation for the N-doped TNAs.

It has been found that the two-step anodization is a good method for preparing much more uniform TNAs [31,41] and the growth of diverse top-layer morphologies covering on TNAs [42]. The TNAs with a grid-like top-layer structure are an outcome of competition between the electric-field-driven anodic oxidation of Ti to form TiO_2_, and the electric-field-assisted chemical dissolution of the TiO_2_ layer [41,47]. The reactions are given below:

Anodic reaction: Ti + 2H_2_O − 4e → TiO_2_ + 4H^+^

Cathodic reaction: 4H^+^ + 4e → 2H_2_

Chemical etching (dissolution) reaction: TiO_2_ + 6F^−^ + 4NH_4_^+^ → TiF_6_^2−^ + 2H_2_O.

The anodic oxidation reaction occurs as Ti^4+^ ejection and deposition on the surface in the form of TiO_2_, while the TiF_6_^2−^ etching reaction occurs from top to bottom of the as-grown TiO_2_. The anodic oxidation rate is very fast and dominated over the NH_4_F etching rate, resulting in a thin oxide layer in the early stage [31,41]. In the late stage, the deposition rate of TiO_2_ at the entrance of nanotubes slows down, while field-induced random dissolution of the surface becomes more significant or dominant to form pore-like structures, which further develop into TNA structures [31,41,47]. At certain relative rates between TiO_2_ deposition and dissolution, a layer of interconnected nanopores can be constructed on the top of TiO_2_, as shown in the inset of Figure 2b. Notably, the first anodization step offers highly ordered imprints after ultrasonication removal, which plays the role of a template for the subsequent growth of well-aligned nanotubes [31]. The highly ordered TNAs are believed to have potential applications in fields such as solar cells, photonic crystals, photocatalyst, and hydrogen storage [31,41,42].

**Figure 3:**
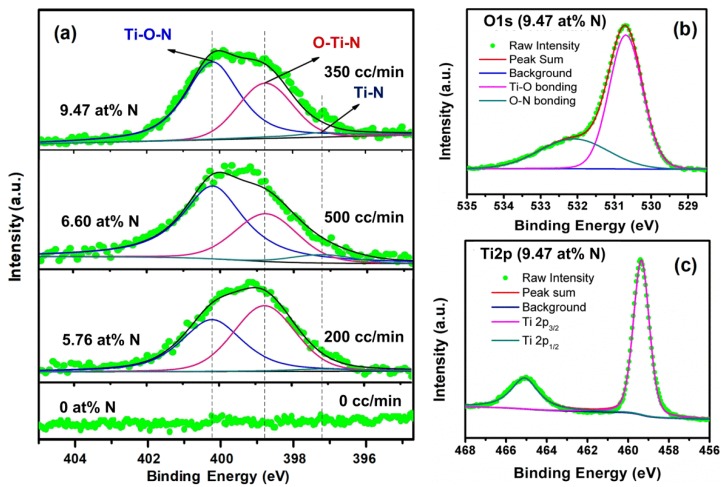
(**a**) XPS spectra of N1s of the pristine and N-doped TNAs. XPS spectra of (**b**) O1s and (**c**) Ti2p of the 9.47 at % N-doped TNAs.

XPS analysis was performed to quantitatively determine the N-doping concentrations for all the TNAs samples. Figure 3a shows the high-resolution XPS N1s core level spectra of the samples. There was no N1s peak for the TNA annealed in a vacuum. Meanwhile, clear N1s peaks appeared in all the TNAs annealed in N_2_ gas flows, indicating the presence of N species on the sample surfaces. The N1s peaks can be fitted well with three components, namely Ti–O–N linkage at 400.2 eV [48,49,50], O–Ti–N linkage at 398.7 eV [50], and Ti–N linkage at 397.1 eV [51]. Figure 3b shows a typical O1s spectrum of N-doped TNAs (i.e., the 9.47 at % N sample). As expected, the Ti–O and O–N bonds were clearly observed from O1s spectra of the N-doped TNAs at 530.4 eV and 532.2 eV, respectively [48,49,50]. Figure 3c presents the two Ti2p peaks: Ti2p_1/2_ at 459.2 eV and Ti2p_3/2_ at 464.9 eV. The XPS results for N-doped TiO_2_ agree well with those in [49,50,52].

The quantitative results of XPS analysis are summarized in Table 1. The N doping concentration varied with the N_2_ gas flows. The N concentration increased from 0 to 9.47 at % with increasing N_2_ gas flow from 0 (vacuum) to 350 cc/min, but it decreased under a sufficient high N_2_ gas flow of 500 cc/min. As shown in Figure 3a, the component of Ti–N monotonically increase with increasing N_2_ gas flows, suggesting the presence of TiN ultra-thin layer on the surfaces of N-TNAs. Thus, it is reasonable to believe that a considerably thick TiN layer was grown during the annealing process at 450 °C under a sufficient high N_2_ gas flow (i.e., 500 cc/min). The TiN layer can constrain the injection of N atoms into TNAs via thermal diffusion, resulting in the lower N-doping concentration for 500 cc/min than it is for 350 cc/min (Table 1).

**Figure 4:**
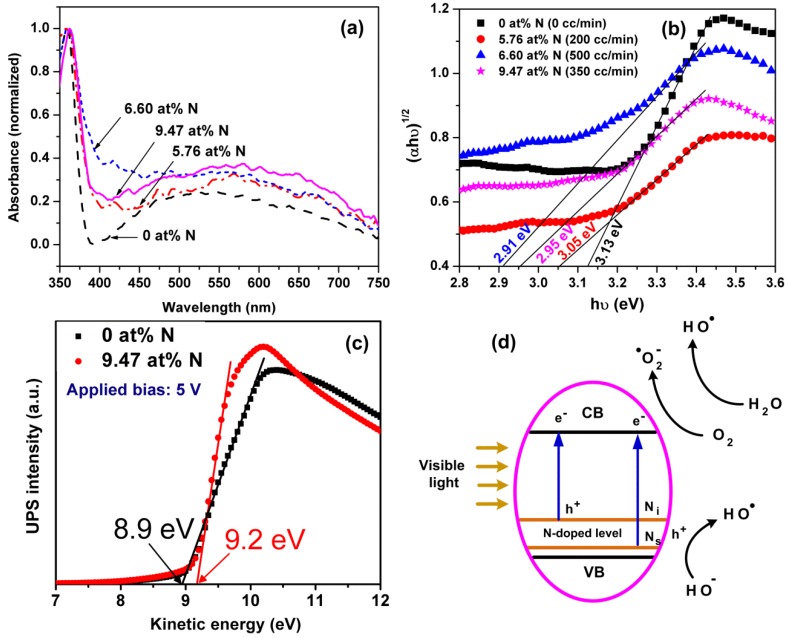
(**a**) UV-Vis absorption spectra of pristine TNAs and N-TNAs with various N-doping levels. (**b**) Plot of (αhν)^1/2^ vs. hν of the samples. (**c**) UPS spectroscopy of pristine TNAs and N (9.47 at %)-TNAs, (**d**) the schematic band diagram of N-TNAs.

The optical absorption is an important property of a photocatalyst. The UV-visible absorption (Figure 4a) is recorded in the range of 350–750 nm. The band gap (*E_g_*) is determined by plotting of (αhν)^1/2^ against the energy (*hν*), and by extrapolating the straight line to *hv* axis, as shown in Figure 4b, where α is the absorption coefficient, *hν* is the photon energy. The *E_g_* of TNAs, with 0, 5.76, 6.60, and 9.47 at % N-TNAs were found to be 3.13 eV, 3.05 eV, 2.91 eV, and 2.95 eV, respectively. The band gap narrowing effect for N-TNAs could be attributed to the presence of N-doped levels (see Figure 4d). It has been reported that N-doping will introduce N2p states near valance band (VB) [53]. Moreover, the incorporation of nitrogen into the TiO_2_ structure occurs via substitutional and interstitial means [11]. The substitutional doping (N_s_) involves oxygen replacement, which reduces the *E_g_* to 3.06 eV [11,54]. Meanwhile, the interstitial doping (N_i_) significantly reduces the *E_g_* to (~2.46 eV) [11,54]. The present slight *E_g_* reduction of N-TNAs suggests that the substitutional N doping is the dominant mechanism.

To further elucidate band structures of the samples, the work functions of a pristine TNAs and a N-TNAs (9.47 at %) are characterized by UPS, and the results are shown in Figure 4c. In the experiments, the applied bias is 5 V, and the kinetic energy is determined by drawing the tangent of the spectra. The intercept of the straight line with *x*-axis are found to be 8.9 eV and 9.2 eV for the TNAs and the N-TNAs, respectively (Figure 4c). Thus, the calculated work functions are 3.9 eV for TNAs and 4.2 eV for the N-TNAs. The UPS results were consistent with the results by Sudhagar et al. [45], where the N-ion implanted TiO_2_ also had larger work function than that of pristine TiO_2_ (i.e., 3.9 vs. 3.7 eV). This reflects the incorporation of N atoms into TiO_2_, which induces the modification of the TiO_2_ lattice through substitutional doping of oxygen with nitrogen and interstitial nitrogen [53,55,56]. Figure 4d shows a schematic band diagram of N-TNAs, in which the N-doping states is located just above the VB, and will involve in photocatalytic activity of N-TNAs in the visible range.

**Figure 5:**
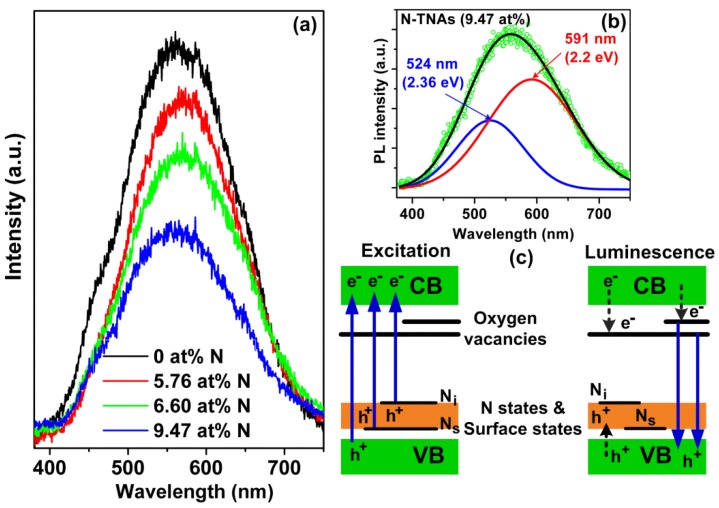
(**a**) Photoluminescence spectra of TNAs and N-TNAs with different N concentrations from 5.76 to 9.47 at %. (**b**) The fitting PL spectrum for the N-TNAs (9.47 at %), two peaks can clearly be observed. (**c**) Energy level model for excitation and luminescence processes in N-TNAs.

The insights into the charge recombination process, the effectiveness of trapping, migration and transfer of charge carriers are revealed by means of PL spectra of the TNAs and N-TNAs. As PL emission mainly results from the recombination of excited electrons and holes, a lower PL intensity may indicate a lower recombination rate of electron‒hole pairs and higher separation efficiency under the same test conditions [57,58]. Figure 5a shows the PL spectra of TNAs (0 at % N) and N-TNAs (5.76–9.47 at % N). Clearly, broad PL peaks are observed around 558 nm (~2.22 eV) for the anatase-phase TNAs and N-TNAs, whose origin has been considered to be partially reduced titanium ions, self-trapped excitons, oxygen vacancies and surface states [35,59]. As shown in Figure 5b, the PL emission spectrum of a N-TNAs (9.47 at %) can be fitted by two peaks at 591 nm (2.2 eV) and 524 nm (2.36 eV). The energy levels of oxygen vacancies are located at ~0.5 eV and ~0.8 eV below the conduction band (CB) of TiO_2_ [60]. Thus, the photogenerated electrons in the CB can fall into the oxygen vacancies through a non-irradiative process, and then they recombine with photogenerated holes in the valence band (VB) to result in the fluorescence emission (see Figure 5c) [60]. Furthermore, as shown in Figure 5a, the PL intensity of N-TNAs systematically decreases with increasing N-doping concentration. The lower PL intensity of N-TNAs can be attributed to the capture of photogenerated holes by surface states (surface nitrogen species) and N states near VB (Figure 5c), agreeing with the PL results in [60]. The PL results further confirm the presence and N states, which serve as a hole reservoir to induce excitation under visible light irradiation and improve electron‒hole separation efficiency.

**Figure 6:**
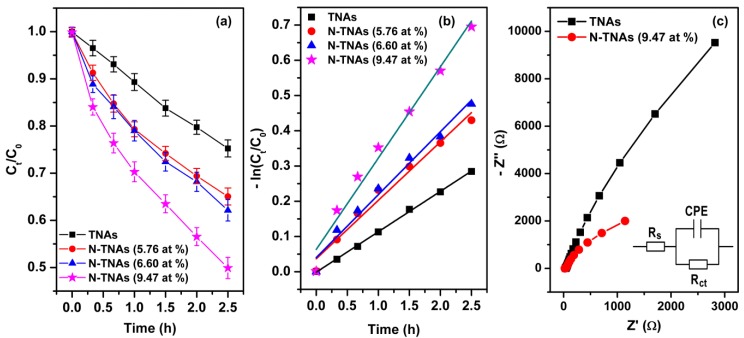
(**a**) Photocatalytic activity of TNAs and N-TNAs recorded in 0.5 ppm methylene blue solution under visible light illumination; the error bar indicates standard error of the mean data of three sample series. (**b**) Methylene blue degradation kinetic curves of TNAs and N-TNAs with different N-doping concentrations under visible light illumination. (**c**) EIS Nyquist plots for a TNA and a N-TNAs (9.47 at %) under visible light irradiation.; the inset is the equivalent fitting circuit.

The photocatalytic activity of TNAs and N-TNAs were studied through photocatalytic degradation of methylene blue (MB) under visible light irradiation. As can be seen in Figure 6a,b, all the N-TNAs possessed higher photocatalytic activity in MB degradation than that of the TNAs, and the activity of N-TNAs increased with increasing N concentration from 5.76 to 9.47 at %. The pseudo-first-order rate constants were determined by fitting the data with the Langmuir–Hinshelwood kinetics rate model [61,62]. The TNAs had a reaction rate constant (k) of 0.115 (h^−1^), and k increased monotonically with increasing N-doping concentration (see Table 1). The N-TNA (9.47 at %) achieved the highest k value up to 0.259 (h^−1^), which was 125% higher than that of the pristine TNAs. The present enhancements in photocatalytic degradation MB for N-TNAs are attributed to the localized nitrogen (N-doping states) that enables N-TNAs to enhance both visible light absorption and electron‒hole separation. A local N inter-band induces the optical absorption of TNAs, which can enhance the generation of electron‒hole pairs. This study demonstrates that the facile thermal in N_2_ ambient at an elevated temperature of 450 °C allows introducing certain N-doping into TiO_2_ lattice to result in the significant enhancement in photocatalytic activity of TiO_2_ nanomaterials.

To investigate electron transport properties of TNAs and N-TNAs at the solid–liquid interface, the electrochemical impedance spectroscopy (EIS) data were collected under room light illumination. Figure 6c shows the EIS Nyquist plots for TNAs and N-TNAs (9.47 at %). Clearly, the circular arc radius of N-TNAs electrode is much smaller than TNAs electrode, indicating the smaller interface charge transfer resistance (*R_ct_*) of the former. Indeed, by fitting with the equivalent circuit in the inset, we yielded *R_ct_* = 19,869 Ω for TNAs and *R_ct_* = 6482 Ω for N-TNAs. Thus, it suggests that the introduction of N atom is more beneficial to the separation of the photo-induced electrons and holes and faster charge transfer than that of the TNAs electrode at the solid–liquid interface, agreeing with the results in [4,22,63].

Table 2 summarizes the synthesis methods of N-doped TiO_2_-based nanomaterials and their photocatalytic performance in degradation various organic pollutants under light irradiation. The photocatalytic degradation rate of the N-TNAs (9.47 at %, k = 0.26 h^−1^) in this study was twice as high than that of N-TNAs (k = 0.11 h^−1^) [22], N-TiO_2_ nanowires (k = 0.13 h^−1^) [63], and CdS–Ag/TiO_2_ nanotubes (k = 0.13 h^−1^) [64]. However, it was almost 2–6 times lower than the k constants of N-TiO_2_ nanoparticles (k = 0.44 h^−1^) prepared by sol-solvothermal process [58], N-TiO_2_ nanosheets (k = 0.45 h^−1^) synthesized chemical route and electrospinning technique [52], and N-TNAs prepared anodic oxidation [4]. It is worthy of mentioning that the difference in k-constant results can come from the differences in treated solution and intrinsic properties of the materials such as surface area, crystallinity, N-doping concentration, band structure, etc. 

## 4. Conclusions

Modified TNAs and N-TNAs were fabricated by a two-step anodization, followed by thermal annealing at 450 °C in different N_2_ gas flows for 3 h. TNAs exhibits highly order uniformly and has a grid-like top layer that is grown by the competition between the electric-field-driven anodic oxidation of Ti to form TiO_2_, and the electric-field-assisted chemical dissolution of the TiO_2_ layer. The XPS, UV-Vis, UPS, and PL results confirm that N atoms are successfully incorporated into TiO_2_ lattice to introduce N states (or N-interband) just above the valance band. The N states induce the narrowing the band gap effect to yield the enhancement of visible light absorption for N-TNAs. In addition, N-states easily trap holes and thus serve as carrier reservoir to provide more photocarriers for enhancing photocatalytic activity under visible light irradiation. It also enables us to reduce the electron–hole recombination rate. Thanks to these synergetic effects, the N-doped TNAs with 9.47 at % N exhibit superior photocatalytic activity in MB degradation with *k* = 0.26 h^−1^, which accounts for a 125% enhancement as compared to the pristine TNAs. The results of this study demonstrate that the simple annealing process is beneficial for introducing N-doping into TiO_2_ and enhanced the photocatalytic activity and applications.

## Figures and Tables

**Table 1:** N atomic concentration and reaction rate constant (*k*) in 0.5 ppm methylene blue solution of the TNA samples annealed in various N_2_ gas flows.

N_2_ Gas Flow (cc/min)	N at % (Determined by XPS)	Reaction Rate (h^−1^) under Visible Light Irradiation
0	0	1.15 × 10^−1^
200	5.76	1.66 × 10^−1^
500	6.60	1.79 × 10^−1^
350	9.47	2.59 × 10^−1^

**Table 2:** N-doped TiO_2_-based nanomaterials, synthesis methods, organic pollutants, N-doping concentration, and reaction rates of the optimal N-TNAs in this study and in the relevant literature.

Photocatalyst	Synthesis Methods	Organic Pollutants	N Concentration (at. %)	Reaction Rate (h^−1^)	Ref.
Modified N-TNAs	Two-step anodization	Methylene blue	9.47	0.26	This study
N-TiO_2_ nanosheets	Chemical route	Methyl orange	-	0.45	[52]
N-TNAs	Anodic oxidation	RhB dye	-	0.11	[22]
N-TNAs	Anodic oxidation	Methyl orange	-	1.62	[4]
N-TiO_2_ nanowires	Hydrothermal method	Methylene blue	-	0.13	[63]
TiO_2_ nanoparticles	Sol-gel	Methylene blue	5–10	-	[23]
CdS–Ag/TiO_2_ NTs	Two-step anodization	Methyl orange	-	0.13	[64]
N-TiO_2_ nanoparticles	Sol-solvothermal process	Methylene blue	-	0.44	[58]
